# The Struggle Between Chimeric Antigen Receptor T-Cell Therapy and Neurological Complications in Acute Lymphoblastic Leukemia Treatment

**DOI:** 10.3390/cimb47050381

**Published:** 2025-05-21

**Authors:** Norwin Kubick, Marzena Łazarczyk, Omar Awad, Michał Ławiński, Jarosław Olav Horbańczuk, Mariusz Sacharczuk, Atanas G. Atanasov, Piotr Religa, Michel Edwar Mickael

**Affiliations:** 1 Department of Biology, Institute of Plant Science and Microbiology, University of Hamburg, Ohnhorststr. 18, 22609 Hamburg, Germany; kubick.norwin@googlemail.com; 2 Institute of Genetics and Animal Biotechnology, Polish Academy of Sciences, Postepu 36A, 05-552 Jastrzebiec, Poland; m.lazarczyk@igbzpan.pl (M.Ł.); m.lawinski@igbzpan.pl (M.Ł.); j.horbanczuk@igbzpan.pl (J.O.H.); m.sacharczuk@igbzpan.pl (M.S.); a.atanasov@igbzpan.pl (A.G.A.); 3 Medical Faculty, Alexanderia University, Champollion Street, Al Mesallah Sharq, Al Attarin, Alexandria 5372066, Egypt; mooomar187@gmail.com; 4 Department of Pharmacodynamics, Faculty of Pharmacy, Medical University of Warsaw, Banacha 1B, 02-091 Warsaw, Poland; 5 Ludwig Boltzmann Institute Digital Health and Patient Safety, Medical University of Vienna, Spitalgasse 23, 1090 Vienna, Austria; 6 Department of Medicine, Karolinska Institute, Visionsgatan 18, 171 76 Solna, Sweden; piotr.religa@ki.se

**Keywords:** CAR T-cell therapy, acute lymphoblastic leukemia, immunotherapy, cytokine release syndrome

## Abstract

Acute lymphoblastic leukemia (ALL) accounts for approximately 25% of childhood cancers and 20% of leukemia cases in adults, with a higher prevalence in males than females. It is characterized by symptoms such as fatigue, fever, and bone pain and poses a significant risk of mortality if left untreated. While chemotherapy and stem cell transplantation are standard treatments, their efficacy declines in relapsed or refractory cases, highlighting the need for innovative therapeutic approaches. CAR T-cell therapy has emerged as a transformative technology, offering the potential to overcome these challenges and deliver durable remissions. CAR T-cell therapy demonstrates significant advantages, including targeting specific antigens, overcoming high-risk genetic mutations, and achieving sustained remissions in both pediatric and adult patients. However, notable challenges remain, such as cytokine release syndrome (CRS) and immune effector cell-associated neurotoxicity syndrome (ICANS). In this review, we focus on neurological symptoms associated with CAR T-cell therapy in treating ALL and discuss current and future strategies aiming at reducing their risk.

## 1. Introduction

Acute lymphoblastic leukemia (ALL) is a hematologic malignancy resulting from the clonal proliferation of lymphoid progenitor cells. Lymphoblasts infiltrate the bone marrow, peripheral blood, and extramedullary sites, disrupting normal hematopoiesis [1]. ALL is the most common leukemia in children, accounting for approximately 75–80% of all childhood leukemia cases [2]. The annual incidence in children is around 3–4 cases per 100,000. In adults, ALL is much less common, comprising only about 15–20% of acute leukemias in this population. The incidence in adults is approximately 1–2 cases per 100,000 per year [3]. B-ALL is driven by various genetic alterations that disrupt normal B-cell development, including chromosomal abnormalities such as hyperdiploidy and hypodiploidy, as well as key translocations like BCR-ABL1 and ETV6-RUNX1, which affect signaling pathways and transcriptional regulation. Other common genetic abnormalities in B-ALL include deletions of IKZF1 and aneuploidy [4]. In T-ALL, genetic alterations primarily affect pathways regulating T-cell differentiation and proliferation, with activating mutations in NOTCH1 driving uncontrolled growth, inactivating mutations in FBXW7 impairing degradation of oncogenic substrates, and translocations involving T-cell receptor loci leading to overexpression of oncogenes like MYC and TLX1 [5]. Manifestations in both B-ALL and T-ALL include anemia (fatigue, pallor, dyspnea), thrombocytopenia (bruising, gum bleeding, nosebleeds), and neutropenia (recurrent infections). Patients may also experience bone pain, particularly in children, as well as organomegaly (splenomegaly, hepatomegaly) and lymphadenopathy, which can be localized or generalized [6]. The overall prognosis of ALL has significantly improved over the past few decades, with survival rates now exceeding 90% in pediatric cases and reaching 40–50% in adults, largely due to advancements in chemotherapy, supportive care, and targeted therapies [7]. However, prognosis varies based on ALL subtypes and age. B-ALL generally has better outcomes than T-cell ALL (T-ALL), particularly in children, where long-term survival for B-ALL exceeds 90%, whereas T-ALL survival rates range from 75 to 85%. In adults, survival remains lower, with B-ALL patients achieving 40–50% long-term survival, while T-ALL cases have a poorer prognosis, typically below 40%.

Despite these advances, a significant subset of patients experience relapsed or refractory (R/R) ALL, posing a major clinical challenge. Relapsed ALL occurs when patients who initially achieve remission experience disease recurrence, while refractory ALL refers to cases that fail to respond to frontline therapy [8,9]. R/R ALL is associated with high mortality due to the emergence of chemoresistant leukemic clones, leading to limited treatment options [10]. The prognosis for these patients is poor, with survival rates dropping below 10–20% in adults and 30–40% in children. Standard salvage therapies, including intensified chemotherapy and hematopoietic stem cell transplantation (HSCT), have limited success, often accompanied by severe toxicities [11]. This unmet clinical need has driven the development of novel immunotherapies, including monoclonal antibodies and chimeric antigen receptor (CAR) T-cell therapy, which have shown promising results in R/R ALL. However, this therapy is not without complications, as immune-related toxicities such as cytokine release syndrome (CRS) and immune effector cell-associated neurotoxicity syndrome (ICANS) remain significant barriers to optimizing patient outcomes [12,13,14]. This review will discuss the challenges posed by neurological and inflammatory symptoms associated with CAR T-cell therapy in the context of ALL, highlighting factors mediating these complications and strategies to mitigate toxicity while preserving therapeutic efficacy.

## 2. CAR T-Cell Therapy Process for ALL

### 2.1. Current Status of CAR T-Cell Therapy

To date, CAR T-cell therapy has passed through four stages of research. The first generation was pioneered by Jensen et al., who designed one of the first CAR T cells to target B-lineage lymphomas by developing a CAR with a single-chain antibody Fv (scFv) that contained the variable regions of both the heavy and light chains, linked flexibly to enable antigen binding. The structure also included an Fc region for enhanced stability and a ζ (zeta) domain from the CD3-ζ signaling chain, which transduced activation signals upon antigen engagement. The group used a plasmid-based electroporation system to genetically modify primary human T lymphocytes, allowing for stable chromosomal integration of the chimeric immunoreceptor construct while circumventing the limitations of viral vector systems [15]. This approach led to the development of scFvFc:ζ CARs specific for the B-cell lineage marker CD19, enabling targeted therapy against B-cell lineage lymphomas.

Second-generation CAR T cells were developed by several research teams, including Kowolik et al. [16]. The first-generation CARs relied solely on antigen-dependent CD3-ζ signaling, which limited full T-cell activation due to the absence of costimulatory signals. To overcome this limitation, Kowolik et al. demonstrated that enforcing the expression of the full-length CD28 costimulatory molecule in CD8^+^CD19R^+^CD28^−^ T cells restored antigen-dependent activation when interacting with CD19^+^ targets expressing CD80/CD86. To provide intrinsic costimulation independent of CD80/CD86 binding, they developed a second-generation CAR (CD19RCD28) incorporating a chimeric CD28 signaling domain fused to CD3-ζ. Compared with CD19R^+^ T cells, CD19RCD28^+^ T cells exhibited superior proliferation without exogenous IL-2, produced IL-2, propagated efficiently, and upregulated the anti-apoptotic protein Bcl-XL after CD19^+^ tumor cell stimulation.

Pan et al. expanded the realm of CAR T-cell therapy by developing specialized CAR T cells targeting CD7, a marker present in T-ALL cells. In a Phase I clinical trial, they infused donor-derived anti-CD7 CAR T cells into 20 patients with relapsed or refractory T-ALL, assessing both safety and efficacy. They found that the treatment led to a high complete remission rate with manageable side effects. Interestingly, while patients’ normal CD7-positive T cells were depleted, CD7-negative T cells expanded, which may have helped mitigate treatment-related immunodeficiency [17].

The third generation was introduced by various groups, including Wilkie et al., who developed a novel CAR T-cell therapy targeting MUC1, a tumor-associated antigen that is overexpressed in most human cancers. They optimized the CAR’s design by incorporating an IgD hinge to overcome steric hindrance and using an scFv from the HMFG2 hybridoma for strong binding across MUC1 glycoforms. The final construct, HOX, featured a CD28/OX40/CD3ζ signaling domain, enhancing T-cell proliferation, cytokine production, and tumor cell killing. In vivo, HOX-expressing T cells significantly delayed tumor growth in a xenograft model [18]. Another third-generation attempt was made by Schultz et al., who introduced a humanized CD22 CAR with a 41BB costimulatory domain using an automated, closed-loop manufacturing system. While it achieved 75% complete remission, durability was limited, with CD22 downregulation contributing to relapse [19].

In the fourth generation, Sringaseh et al. introduced Cytokine Support for CAR-T Persistence. This research team combined CD19/CD22 CAR T therapy with NKTR-255, an IL-15 receptor agonist, increasing CAR T persistence and improving progression-free survival while enhancing lymphocyte trafficking to tissues, including the CNS [20]. Also, Feng et al. developed CD5 CAR T cells incorporating an IL-15–IL-15sushi complex to target T-cell malignancies. Their approach rapidly cleared CNS lymphoblasts in a refractory T-LBL patient, achieving complete remission with only transient T-cell aplasia, demonstrating promise in treating T-cell leukemias with CNS involvement [21]. Also, in the context of T-ALL, Chisea et al. used base editing to inactivate three genes encoding CD52 and CD7 receptors and the β chain of the αβ T-cell receptor to evade lymphodepleting serotherapy, CAR7 T-cell fratricide, and graft-versus-host disease, respectively. However, the sample size consisted only of two patients; thus, it is difficult to judge its effectiveness [22].

CAR T cell therapy is progressing with novel approaches emerging rapidly. Overall, three main methods are currently used to control CAR T cells. Passive control methods involve affinity-tuned CARs and the transient transfection of T cells to regulate their activity. Inducible control strategies include eliminating CAR T cells with antibodies or using inducible suicide systems as well as employing drugs to manage CAR expression either at the transcriptional level or by assembling split-CARs with separated extra- and intracellular domains. Another approach involves decoupling the binding domain from the intracellular signaling domain, allowing for the use of binding adapters that can be adjusted as needed. In contrast, autonomous CAR T cells are designed to self-regulate, and deciding whether to initiate or withhold cytotoxic activity is based on the surface proteins of healthy and cancerous cells [23,24].

### 2.2. Complications of CAR T-Cell Therapy

Early clinical trials identified CRS as a significant challenge in CAR T-cell therapy, with toxicities ranging from fever and hypotension to life-threatening complications [25]. Brentjens et al. demonstrated that low-dose CD19-specific CAR T cells were generally well tolerated, but one patient who received lymphodepleting chemotherapy before infusion developed severe toxicity, including hypotension, dyspnea, and renal failure, leading to death [26]. Kochenderfer et al. found that anti-CD19 CAR T-cell therapy, following chemotherapy and IL-2 administration, induced remissions in most patients, but elevated IFNγ and TNF levels were linked to acute toxicities [15]. Porter et al. observed that CD19-specific CAR T cells incorporating CD137 and CD3-zeta expanded over 1000-fold in vivo, achieving complete remission, though hypogammaglobulinemia emerged as a chronic side effect [27]. Severe cytokine release syndrome remains a major challenge in CAR T-cell therapy for B-ALL and T-ALL. Wang et al. reported that, among 225 pediatric patients treated with CD19/CD22 CAR T cells, 88% developed CRS, with 20.9% experiencing neurotoxicity and three fatal cases of severe CRS, highlighting its lethal risks. Despite a 12-month event-free survival (EFS) of 73.5%, relapse due to antigen escape remained a significant concern. Similarly, Tan et al. found that donor-derived CD7 CAR T cells achieved a 95% response rate in refractory or relapsed T-ALL, with 85% reaching complete remission. However, 30% relapsed within six months, primarily due to CD7 antigen loss. Long-term follow-up revealed a progression-free survival (PFS) rate of 36.8% and an overall survival (OS) rate of 42.3%, with late complications such as severe infections and Grade 4 graft-versus-host disease underscoring the need for improved toxicity management and strategies to prevent antigen escape [28].

CRS symptoms are not the only complications associated with CAR T-cell therapy. Park et al. found that CAR T-cell infusion also carries a significant risk of infection. In a study of 53 adult patients with relapsed B-ALL, 42% experienced 26 infections within the first 30 days post-infusion. Additionally, among 32 patients who achieved complete remission, 31% developed infections between days 31 and 180, with respiratory viruses being the most common late-stage infections. The severity of the CRS significantly increased the infection risk, particularly bloodstream infections, with three patients dying due to infection-related causes [12].

Additionally, Prudent et al. found that neuropsychiatric toxicity was another significant complication. This group of researchers reported two cases of CAR-T-related neurotoxicity. One patient experienced mild delirium and aphasia, which resolved without intervention, while the other developed severe delirium, seizures, and respiratory insufficiency requiring intensive care. These cases highlight the variability in neurotoxicity severity, ranging from transient cognitive impairment to life-threatening complications [29].

Although ICANS is typically reversible, some patients exhibit long-term neurocognitive deficits, including memory impairment, executive dysfunction, and reduced processing speed, particularly those who experienced severe or prolonged neurotoxicity during treatment. The exact mechanisms are unclear but may involve cytokine-mediated blood–brain barrier disruption, neuronal damage, or persistent neuroinflammation. Long-term CRS complications have been extensively reported and they include neutropenia, thrombocytopenia, and anemia, which often persist for weeks to months post-infusion [30]. Also, since CAR T cells targeting CD19 also deplete normal B cells, many patients experience prolonged B-cell aplasia, leading to chronic hypogammaglobulinemia and increased susceptibility to bacterial infections. Additionally, the persistence of CAR T cells may delay normal B-cell reconstitution, affecting long-term immune competence and vaccine responses [31]. Additionally, prolonged immune activation and chronic inflammation post-CAR T-cell therapy could contribute to a pro-tumorigenic environment, warranting long-term monitoring and genetic surveillance in patients undergoing treatment. While these risks remain rare, they highlight the need for safer gene-editing techniques, such as CRISPR-based approaches, to reduce the likelihood of oncogenic transformation [32,33]. However, the long-term relationship between CRS and ICANS has not yet been fully investigated.

### 2.3. Clinical and Therapeutic Risk Factors of Immune Effector Cell-Associated Neurotoxicity Syndrome

Long-term analysis and refractory studies revealed that many factors contribute to the outcome of CAR T-cell-based therapy. One of the factors is the disease burden. Park et al. conducted a long-term clinical trial with follow-up. They treated 53 adults with relapsed B-ALL using 19-28z CAR T cells and observed an 83% complete remission rate. However, 26% of the patients developed CRS with significant neurotoxic events and one patient died. At a median follow-up of 29 months, the median event-free survival was 6.1 months, and the median overall survival was 12.9 months. Patients with a low disease burden had significantly longer survival and lower toxicity, while those with a higher disease burden had worse survival and more CRS and neurotoxic events. These findings suggest that early treatment with CAR T cells, before the disease burden increases, may improve long-term outcomes and reduce toxicity [34].

### 2.4. Molecular and Mechanistic Contributors to Immune Effector Cell-Associated Neurotoxicity Syndrome

A higher disease burden in CAR T-cell therapy is strongly linked to neurological symptoms due to excessive cytokine release and immune activation [35]. Patients with a greater tumor load often experience more robust CAR T-cell expansion, leading to elevated levels of inflammatory cytokines such as IL-6, TNF-α, and IFN-γ (Table 1) [36]. This excessive cytokine release increases the blood–brain barrier (BBB)’s permeability, allowing inflammatory mediators to enter the central nervous system (CNS) and contribute to neuroinflammation [37]. As a result, patients with a high disease burden are at a greater risk of developing immune effector cell-associated neurotoxicity syndrome. Additionally, endothelial activation and coagulation abnormalities, including increased von Willebrand factor (VWF) levels, can exacerbate neurological dysfunction by promoting microthrombosis in the brain [38,39,40]. Overall, these factors highlight the importance of early CAR T-cell therapy intervention before the disease burden escalates, as it may reduce neurotoxicity and improve long-term outcomes.

Genetic factors influence CAR T-cell therapy neurological outcomes. An et al. conducted a Phase II clinical trial on CD19 CAR T cells in 51 R/R B-ALL patients, reporting an 80.9% overall remission rate. In general, high-risk genetic factors such as Philadelphia chromosome (Ph)+ ALL, Ph-like ALL, t (v;11q23), hypodiploidy, and complex karyotypes (≥5 abnormalities) were associated with worse OS and RFS, identifying them as independent risk factors for poor CAR T-cell outcomes [41]. Ph+ and Ph-like ALL drive continuous tyrosine kinase activation, leading to heightened cytokine release, particularly IL-6 and IFN-γ, which exacerbates CRS and ICANS [42,43]. MLL rearrangements (t (v;11q23)) are linked to severe monocyte/macrophage activation, increasing CRS severity and the risk of CNS infiltration, which can directly worsen neurological symptoms [44]. Hypodiploid ALL, characterized by genomic instability and therapy resistance, could trigger excessive inflammatory responses and prolong disease persistence, further increasing neuroinflammation. However, a direct relationship has not been found. Similarly, complex karyotypes (≥5 chromosomal abnormalities) result in immune dysregulation, leading to prolonged CAR T-cell persistence and excessive cytokine release. We anticipate that this abnormality could be linked to CNS inflammation; however, direct evidence is still lacking. Collectively, these genetic factors seem to contribute to an increased neurotoxicity risk by increasing the pro-inflammatory response, which, in turn, increases the vulnerability of the CNS.

Autologous CAR T-cell therapy remains the gold standard due to its superior efficacy and lower risk of neurological symptoms. In contrast, allogeneic CAR T-cell therapy is emerging as an option for patients ineligible for autologous therapy. Del Bufalo et al. demonstrated that donor-derived CAR T cells could induce complete remission in heavily pretreated BCP-ALL patients who relapsed post-allo-HSCT, with eight of thirteen patients maintaining remission at 12 months. However, allogeneic therapy poses a higher risk of immune rejection and graft-versus-host disease (GVHD), leading to increased CNS inflammation. Donor-derived T cells are more likely to cause excessive cytokine release and greater BBB permeability, which elevates the risk of neurological complications. A meta-analysis by Anagnostou et al. reported an 83% remission rate for autologous CAR T cells compared with 55% for allogeneic CAR T cells [43]. The use of patient-derived T cells enhances therapeutic effectiveness, likely due to better persistence, lower immunogenicity, and a reduced risk of GVHD. This approach also minimizes systemic inflammation, thereby decreasing the incidence of severe CRS and immune effector cell-associated neurotoxicity syndrome (ICANS). Despite a comparable toxicity profile between the two approaches, the long-term durability of allogeneic CAR T-cell therapy remains uncertain [45,46].

Prior drug exposure, particularly fludarabine, cyclophosphamide, and inotuzumab ozogamicin (InO), can significantly impact neurological outcomes in CAR T-cell therapy. Shah et al. investigated CD19 CAR T-cell therapy in children and young adults (CAYAs) with relapsed or refractory B-ALL and found that, among 50 patients, 62% achieved complete remission, with 90.3% being MRD-negative [47]. Notably, prior exposure to fludarabine and cyclophosphamide (Flu/Cy) was associated with higher remission rates but potentially correlated with a lower incidence of severe neurotoxicity. However, no explicit clustering of patients based on Flu/Cy intake was presented. In contrast, Aldoss et al. conducted a retrospective study on 189 adult ALL patients receiving brexucabtagene autoleucel (brexucel) and found that prior inotuzumab ozogamicin (InO) exposure was linked to inferior outcomes, likely due to a higher disease burden and inflammation. While InO is frequently used in R/R ALL for its high efficacy, prior studies have suggested that InO bridging therapy may impair CAR T-cell persistence, elevate cytokine release, and increase the risk of ICANS. However, a definitive causal relationship remains unproven, highlighting the need for further research to optimize sequencing strategies to minimize neurological complications [48,49].

The neurological implications of prior stem cell transplantation on CAR T-cell therapy outcomes present several important considerations. Jacoby et al. observed that patients who underwent allogeneic hematopoietic stem cell transplantation (allo-HSCT) following CAR T-cell therapy did not experience CNS relapse, suggesting a potential protective effect against neurological disease recurrence in CNS-involved B-cell precursor acute lymphoblastic leukemia (BCP-ALL) [50]. Conversely, 41BB-based CAR constructs were associated with higher rates of CNS relapse, indicating a specific neurological vulnerability with certain CAR designs. Jensen et al. reported limited persistence of CAR+ cytotoxic T lymphocytes (CTLs) when administered post-stem cell transplantation, which could impact long-term neurological disease control [51,52]. Daniel Lee et al. found no significant impact of prior stem cell transplantation on the anti-leukemic activity of CD19 CAR T-cell therapy in the CNS, though their findings are complicated by the concurrent use of fludarabine and cyclophosphamide conditioning, which themselves have neurological effects [53]. While allo-HSCT may offer additional protection against CNS relapse, the complete neurological implications of integrating stem cell transplantation with CAR T-cell therapy remain undetermined and require further neurological-outcome-focused studies.

Several molecular pathways have been also implicated in mediating neurological complications in CAR T-cell-based therapy for ALL. Teachey et al. identified specific biomarkers associated with neurological toxicities in CAR T-cell therapy for relapsed/refractory ALL. Their analysis of 51 patients revealed that IFNγ, sgp130, and IL1Ra serve as early predictors of severe CRS, which often manifests with neurological symptoms. In pediatric patients specifically, IFNγ, IL13, and MIP1a demonstrated exceptional predictive value for toxicity development [54,55,56]. The study documented elevated levels of 24 cytokines—including IL8, sIL2Ra, GM-CSF, and MCP1—that correlated with severe CRS and its associated neurological complications. The cytokine profile resembled macrophage activation syndrome (MAS)/hemophagocytic lymphohistiocytosis, conditions with known neurological manifestations. Chen et al.’s Phase I trial of CD7 CAR T cells in T-ALL/LBL patients demonstrated complete remission in all seven participants but revealed significant neurological complications. Most notably, one patient died from brain hemorrhage, a severe neurological adverse event directly attributable to the therapy. The study identified potential biomarkers, including S100A8 and S100A9, which may help predict both relapse and neurological complications, providing valuable insights for future optimization of CAR T-cell therapies to mitigate neurological risks [57].

Neurofilament light chain (NfL) has emerged as a promising biomarker of neuronal injury and neurotoxicity, including in the context of immune effector cell-associated neurotoxicity syndrome (ICANS) [58]. Recent studies have shown that plasma NfL levels are significantly elevated in patients experiencing ICANS, particularly in severe cases, suggesting a correlation between NfL concentration and the extent of neurotoxicity [59]. Importantly, elevated baseline NfL levels—prior to CAR T-cell infusion—have been associated with an increased risk of subsequent ICANS development, highlighting its potential as a predictive marker [60]. Elevated NfL levels detected prior to CAR T-cell therapy have been linked to an increased likelihood and severity of subsequent ICANS, suggesting the presence of underlying neuroaxonal damage. This association points to serum NfL as a potential early biomarker for identifying patients at higher risk of developing severe neurotoxicity following treatment. Incorporating NfL measurements into pretreatment assessments could enhance patient monitoring strategies. Nonetheless, these findings require confirmation through larger, prospective clinical studies [61].

The onset of ICANS following CRS suggests a mechanistic continuum rather than distinct, isolated toxicities. Cytokine release during CRS—particularly elevated IL-6, IL-1, and IFN-γ—has been shown to compromise the integrity of the blood–brain barrier (BBB), facilitating neuroinflammatory responses [62]. IL-6 plays a pivotal role in this process by promoting endothelial activation and increasing vascular permeability, which allows cytokines and immune effector cells to infiltrate the central nervous system (CNS) [63]. This cascade could trigger glial cell activation, neuronal injury, and the clinical manifestations of ICANS. Moreover, the co-elevation of systemic cytokines in both CRS and ICANS supports a shared pathophysiologic axis, where the intensity and duration of systemic inflammation directly influence the likelihood and severity of neurotoxicity [64]. Understanding this interdependence is critical for developing targeted interventions that interrupt the CRS–ICANS progression, such as IL-6 inhibitors or early BBB-protective strategies.

Antigen loss mechanisms contribute significantly to the neurological cytotoxicity observed in CAR T-cell therapy. CD19 splice variants, which produce truncated proteins lacking the extracellular domain targeted by CAR T cells, can lead to treatment failure and persistent disease affecting the central nervous system [65]. This mechanism allows leukemic cells to continue proliferating in neurological sanctuaries while evading immune surveillance. Loss-of-function mutations resulting in complete CD19 depletion similarly compromise CAR T-cell therapy efficacy, potentially allowing for leukemic infiltration of the central nervous system and contributing to neurological complications. The persistence of these antigen-negative cells in the cerebrospinal fluid can cause increased neurological manifestations despite peripheral disease control.

Lineage switching, whereby B-ALL cells transdifferentiate into CD19-negative myeloid leukemia, renders them “invisible” to CD19-directed CAR T-cell therapy [66,67]. This phenomenon has been associated with increased rates of CNS relapse and neurological toxicity due to the loss of targeted therapy effectiveness in neurological compartments.

Similarly, the CD22 downregulation observed in patients receiving CD22-directed CAR T-cell therapy demonstrates another escape mechanism that may contribute to neurological disease progression and associated symptoms, as CD22-negative cells can continue to proliferate within the CNS despite the presence of CAR T cells [68].

T-cell exhaustion during CAR T-cell therapy for acute lymphoblastic leukemia (ALL) could contribute to specific neurological manifestations that can complicate treatment. When CAR T cells experience prolonged antigen stimulation, they upregulate exhaustion-associated transcription factors, including TOX, NR4A, and BATF, leading to a dysfunctional state that impacts neurological outcomes. Zebeley et al. demonstrated that CAR T cells seem to rapidly transition toward exhaustion after infusion, with widespread erasure of repressive DNA methylation at effector-related genes coupled with suppression of memory-associated genes like TCF7 and LEF1 [69]. This exhaustion-related reprogramming correlates with reduced cytokine production and impaired cytotoxicity, particularly affecting the clearance of leukemic cells from the central nervous system. The neurological implications of this exhaustion include: (i) an increased risk of CNS relapse due to inadequate clearance of leukemic cells from neurological compartments, (ii) exacerbation of neurotoxicity through dysregulated cytokine production and inflammatory responses, and (iii) prolongation of existing neurological symptoms as exhausted CAR T cells fail to eliminate disease reservoirs. The metabolic stress associated with CAR T-cell exhaustion further contributes to neurological complications. Persistent activation depletes mitochondrial reserves and disrupts glycolysis, leading to an energy insufficiency that particularly affects neurotoxicity management [70]. Mitochondrial dysfunction, increased reactive oxygen species (ROS) production, and impaired oxidative phosphorylation weaken CAR T-cell persistence, correlating with higher rates of neurological adverse events. While CAR T cells engineered to overexpress c-Jun have shown enhanced expansion and anti-tumor potency in mouse models, clinical trials assessing their impact on neurological symptoms are still pending [70,71]. Thus, it is important to note that, while it is theoretically possible that diminished CNS anti-leukemic activity could allow leukemic cells to persist and contribute to neurotoxicity, there is currently limited direct evidence supporting this in the literature.

These previously discussed factors are unlikely to act in isolation but rather interact to influence CAR T-cell therapy neurological complications. For instance, Santomasso et al. analyzed 53 adult patients treated with CD19-specific CAR T cells and found that severe neurotoxicity was linked to multiple converging factors, including high pretreatment disease burden, greater CAR T-cell expansion, and early elevation of proinflammatory cytokines. Notably, while blood–CSF barrier disruption correlated with neurotoxicity severity, it was not associated with CSF white blood cell count or the presence of CAR T cells. Instead, increased levels of proinflammatory cytokines (IL-6, IL-8, MCP1, and IP10) in the CSF suggested a CNS-specific inflammatory response, highlighting the complex interplay between systemic and localized immune reactions in determining CAR T-cell therapy toxicity and efficacy [72].

**Table 1:** Key factors affecting neurological disorders after CAR T-cell therapy.

Factor	Key Outcomes/Implications	References
Disease Burden	Early CAR T therapy may improve long-term outcomes and reduce toxicity.	[34,35,36,37,38,39,40]
Cytokine Release and Neurotoxicity	Increased risk of ICANS, neurological dysfunction, microthrombosis (VWF elevation).	[35,36,37,38,39,40]
Genetic Factors	Linked to higher cytokine release, CRS severity, CNS infiltration, and possible neuroinflammation.	[41,42,43,44]
Autologous vs. Allogeneic CAR T Therapy	Autologous preferred for better persistence, less systemic inflammation, and reduced neurotoxicity.	[43,45,46]
Prior Drug Exposure	Careful sequencing of therapies could optimize outcomes and minimize neurotoxicity.	[47,48,49]
Prior Stem Cell Transplantation	Protective effect possible, but neurological impacts unclear and need more research.	[50,51,52,53]
Cytokine Biomarkers for Neurotoxicity	Cytokine monitoring could predict and mitigate neurotoxicity.	[54,55,56]
New Biomarkers (S100A8/S100A9)	Potential early predictors for relapse and neurological toxicity.	[57]
Neurofilament Light Chain (NfL)	NfL can be used as an early biomarker for identifying at-risk patients.	[58,59,60,61]
CRS–ICANS Continuum	CRS severity directly affects ICANS risk; interventions should target this cascade.	[62,63,64]

### 2.5. Options for Reducing CAR T-Cell Complications

Corticosteroids and IL-6 receptor blockade play crucial roles in managing CRS and ICANS associated with CAR T-cell therapy, though their effects on cytotoxicity differ significantly. Davila et al. identified serum C-reactive protein (CRP) as a reliable biomarker for CRS severity, facilitating early intervention with corticosteroids or IL-6 receptor blockade [73,74]. While IL-6 blockade effectively reduces CRS without direct immunosuppression, its long-term effects on CAR T-cell function remain unclear. In contrast, steroids are known to eliminate CAR T cells, potentially compromising treatment efficacy [75,76,77]. Clinical trials have demonstrated that both steroids and tocilizumab (IL-6 blockade) can reverse inflammatory toxicities associated with CAR T-cell therapy. In a Phase I trial, Davila et al. showed that inflammatory symptoms such as fever, hypotension, and neurotoxicity in patients with relapsed B-ALL were reversible with either corticosteroids or tocilizumab, with the therapy achieving high response rates [78]. Similarly, Daniel et al. found that tocilizumab successfully reversed Grade 4 CRS in pediatric patients, demonstrating its effectiveness in severe cases [79]. Brentjens et al. reported that CRS severity correlated with tumor burden but was effectively managed with steroids, though one patient relapsed due to CAR T-cell loss [80,81]. The timing of corticosteroid administration remains a critical factor in preserving CAR T-cell function. Early dexamethasone use has been shown to mitigate neurotoxicity (ICANS) without significantly impairing CAR T-cell expansion, whereas delayed steroid intervention in severe cases may have a greater negative impact due to prolonged cytokine-driven inflammation. These findings emphasize the importance of optimizing timing and dosage strategies to balance toxicity management while maintaining CAR T-cell efficacy. Grading systems for CAR T-cell complications, such as CRS and ICANS, are essential for standardized diagnosis and management. The American Society for Transplantation and Cellular Therapy (ASTCT) criteria classify these toxicities into four grades based on severity, guiding treatment decisions to balance toxicity control with CAR T-cell efficacy (Table 2).

To manage CRS and neurotoxicity while maintaining efficacy, several studies have explored different CAR T-cell infusion doses. Pan et al. tested doses ranging from 0.05 to 14 × 10^5^/kg in patients with refractory or relapsed B-ALL, later standardizing to 1 × 10^5^/kg. This dose led to a 90% complete remission (CR) or CR with an incomplete count recovery (CRi) rate, with most patients experiencing only mild to moderate CRS [82]. Similarly, Schultz et al. investigated two dose levels (1 × 10^6^ CAR T cells/kg and 3 × 10^6^ CAR T cells/kg) using a bispecific CAR T-cell construct targeting both CD19 and CD22. While the higher dose increased the incidence of severe CRS and ICANS, both were manageable, with an overall response rate of 92% [83]. Frey et al. compared single high-dose infusions to fractionated dosing over three days, finding that fractionated dosing reduced mortality and severe CRS while maintaining a 90% CR rate [84]. In the ZUMA-3 trial, Sha et al. tested different doses of CAR T cells and found that, while higher doses increased the risk of CRS and neurologic events, an optimized dose of 1 × 10^6^ cells/kg balanced safety and efficacy, achieving an 83% complete remission rate [85]. These findings highlight the importance of dose optimization to enhance CAR T-cell therapy outcomes while minimizing severe toxicities.

### 2.6. Cutting-Edge Developments

CAR T-cell therapy has revolutionized treatment for hematologic malignancies, but immune effector cell-associated neurotoxicity syndrome (ICANS) remains a major complication. To address this, a wide range of innovative strategies are being developed across multiple fronts, including CAR design optimization, immune modulation, and patient-specific interventions. Toxicity-reducing CAR constructs such as tunable CARs activated by small molecules and logic-gated CARs recognizing multiple antigens have shown promise in enhancing specificity and minimizing off-target effects [86,87,88,89]. Additionally, alternative cell therapies such as NK or γδ T-cell-based CARs may offer inherently lower neurotoxicity profiles, opening new avenues for safer immunotherapies [90].

Inducible systems for CAR T-cell therapy offer the ability to turn the activation of CAR T cells on and off, providing reversible control over their functions to enhance safety and efficacy. One example is the use of chemical inducers of dimerization (CIDs), which regulate CAR T-cell activity based on dose and timing, such as the MyD88/CD40 signaling domains that enhance anti-tumor effects in solid tumors [91]. Additionally, dietary molecules like resveratrol (RES) have been used in regulation devices to control CAR T-cell activity, with RES-titratable mechanisms allowing for precise modulation of CAR expression [92]. Another approach involves light-inducible systems, where light-triggered dimerization activates CAR expression, offering another layer of control over CAR T-cell activation [93]. These systems ensure that CAR T cells remain inactive in the absence of the trigger and are activated only when needed, preventing unnecessary toxicity. The flexibility of these inducible systems, using both chemical and physical triggers, provides significant potential for improving CAR T-cell therapies.

Gene circuits using Boolean logic gates, such as AND and NOT gates, are employed to control the activation of CAR T cells in a more precise and safe manner. AND-gated CAR T cells require the recognition of at least two tumor antigens to be activated, improving specificity for tumor cells. An example of this system is synNotch, where CARs are activated only if the synthetic Notch receptor (synNotch) recognizes a second antigen [94]. However, off-target toxicity can occur, as seen in the synthetic membrane proteolysis receptor (SNIPR) system, where normal cells may be targeted if they express the wrong antigen. To address this, LINK CARs use a dual-antigen AND-gate approach to ensure only double-positive tumor cells are targeted, reducing collateral damage [95].

On the other hand, NOT-gated CAR T cells combine activating CARs with inhibitory CARs that target ligands on non-tumor cells [96,97]. This safety mechanism inhibits CAR T-cell activity when encountering non-tumor cells, as seen in systems like dual-targeted NOT-gated CAR natural killer (NK) cells, which protect NK cells from fratricide. Other therapeutic cells, such as HEK-293T cells (human embryonic kidney cells) and mesenchymal stem cells (MSCs), can also be engineered with gene circuits to sense disease signals and produce therapeutic effects. The JAK–STAT pathway is often activated to trigger therapeutic gene expression [98,99]. Finally, the protease-based rapid protein secretion system (PASS) allows for quick activation of apoptosis in target cells by releasing cytotoxic proteins like granzyme B and perforin [100]. These systems help overcome delays in therapeutic response by speeding up protein secretion and action.

γδ T cells primarily develop in the thymus, where they generate their γδ T-cell receptor through the process of V(D)J recombination. After specific gene rearrangements, two T-cell lineages, one expressing γδ receptors and the other αβ receptors, emerge from a common lymphoid precursor (CLP). T cells with γδ receptors transmit TCR signals via associated CD3 complexes. While γδ T cells make up 1–10% of circulating T cells in the peripheral blood of healthy adults, αβ T cells dominate, comprising about 90% of circulating T cells, and also transduce signals through CD3 complexes. Unlike αβ TCRs, γδ TCRs can bind directly to antigens without needing MHC molecule presentation, which is why CD4 and CD8 markers are rarely found on γδ T cells. One key characteristic of γδ T cells is their predominant localization in epithelial and mucosal tissues [101]. However, the ligands recognized by γδ T cells are still not fully understood.

γδ T cells possess several superior features compared with classical CAR T cells. First, γδ T cells engineered with CARs targeting specific antigens, such as GD2 or CD19, have demonstrated potent anti-tumor effects, including IFN-γ secretion and tumor cell cytotoxicity in both in vitro and in vivo models. Secondly, Hua et al. demonstrated that blood-derived γδ T cells could adopt a classical regulatory phenotype, expressing FoxP3, CD25, and CTLA-4 and secreting IL-10 and TGF-β, which suppresses CD4+ T-cell proliferation [102]. These suppressive γδ T cells were identified as a distinct subtype expressing Vδ1. Additionally, Traxlmayr et al. showed that Vγ9Vδ2 T cells, while typically anti-tumor, could acquire inhibitory functions in response to IL-12 secreted by DCs, suggesting that their activity can be regulated by negative feedback mechanisms [103,104]. Two main strategies for using γδ T cells in cancer immunotherapy involve adoptive transfer of ex vivo-expanded γδ T cells and in vivo stimulation with phosphoantigens or aminobisphosphonates along with low-dose IL-2. Researchers have developed various protocols to expand γδ T cells, such as using anti-CD2 monoclonal antibodies to produce large numbers of functional γδ T cells, which retain anti-tumor activity. These methods have shown promise in enhancing γδ T cell expansion and generating large quantities for clinical applications, including the development of CAR γδ T cells [104]. On the other hand, γδ T cells exhibit a preference for homing to mucosal and epithelial tissues, which plays a key role in their effectiveness for tumor surveillance. These cells express specific adhesion molecules and chemokine receptors, such as CCR6 and αEβ7, which enable them to efficiently infiltrate mucosal tumors, such as those in the skin or gastrointestinal tract. Thus, it remains unclear whether γδ T cells can be as effective in blood-based cancers, like acute lymphoblastic leukemia (ALL), due to differences in trafficking to tumor sites.

## 3. Conclusions

While CAR T-cell therapy has transformed the treatment landscape for R/R ALL, its success is tempered by significant complications that limit its broader application. CRS and ICANS remain major toxicities, with severity often correlating with disease burden, CAR T-cell expansion, and inflammatory cytokine levels. Additionally, prolonged cytopenias, opportunistic infections, and B-cell aplasia contribute to long-term morbidity, raising concerns about immune recovery and patient quality of life. Multiple factors influence the incidence and severity of these complications, including prior treatment regimens, genetic predispositions, antigen escape, and T-cell exhaustion. The dynamic interplay between tumor burden, host immune environment, and CAR T-cell persistence further complicates toxicity prediction and management. Although advances such as dual-antigen targeting, inducible safety switches, and refined dosing strategies aim to mitigate these risks, balancing therapeutic efficacy with safety remains a formidable challenge. Despite progress, overcoming these barriers requires continued refinement of CAR T-cell engineering, improved biomarker-driven toxicity prediction, and the development of more precise interventions to modulate immune responses. Addressing T-cell exhaustion and antigen loss will be critical for sustaining remissions, while optimizing pre- and post-infusion management strategies may help minimize adverse events. Future research must also focus on enhancing long-term safety, particularly regarding the risk of secondary malignancies and prolonged immune suppression. Ultimately, the promise of CAR T-cell therapy in ALL hinges on our ability to surmount these challenges. A deeper understanding of the biological mechanisms underlying treatment-related complications, coupled with innovative therapeutic strategies, will be essential to making CAR T-cell therapy safer, more durable, and accessible to a broader patient population.

## Figures and Tables

**Table 2:** Grading and Management of CRS and ICANS Based on ASTCT Criteria.

Cytokine Release Syndrome Grading and Management			
Grade	Clinical Presentation	Key Features	Management
Grade 1	Mild symptoms (fever, fatigue)	No hypotension or hypoxia	Supportive care (fluids, antipyretics)
Grade 2	Moderate symptoms	Hypotension responsive to fluids or low-dose vasopressors, mild hypoxia	Tocilizumab ± corticosteroids, IV fluids, oxygen if needed
Grade 3	Severe symptoms	Requires high-dose vasopressors, severe hypoxia requiring oxygen	Tocilizumab + corticosteroids (dexamethasone/methylprednisolone), ICU care
Grade 4	Life-threatening symptoms	Shock requiring multiple vasopressors, mechanical ventilation	High-dose corticosteroids, IL-6 blockade (tocilizumab, siltuximab), ICU care
**ICANS (Neurotoxicity) Grading and Management**			
Grade 1	Mild confusion, attention deficits	No impact on daily function	Supportive care, close monitoring
Grade 2	Moderate cognitive impairment, speech issues	Interferes with daily activities but no life-threatening symptoms	Dexamethasone 10 mg every 6 h
Grade 3	Severe confusion, seizures, motor impairment	Requires hospitalization	High-dose steroids (methylprednisolone 1g/day), ICU if needed
Grade 4	Life-threatening symptoms	Coma, cerebral edema, status epilepticus	ICU care, aggressive steroid therapy, neuroprotective strategies
